# Dengue Virus Serotype 1 Effects on Mosquito Survival Differ among Geographically Distinct *Aedes aegypti* Populations

**DOI:** 10.3390/insects15060393

**Published:** 2024-05-28

**Authors:** Milan S. G. Keirsebelik, Mariana R. David, Márcio Galvão Pavan, Dinair Couto-Lima, Miriam Palomino, Rafi Ur Rahman, Ary A. Hoffmann, Ana C. Bahia, Guy Caljon, Rafael Maciel-de-Freitas

**Affiliations:** 1 Laboratório de Transmissores de Hematozoários, Instituto Oswaldo Cruz, Fiocruz, Rio de Janeiro 21040-900, Brazil; milankeirsebelik96@gmail.com (M.S.G.K.); maridavid@ioc.fiocruz.br (M.R.D.); mgpavan@ioc.fiocruz.br (M.G.P.); dcouto@ioc.fiocruz.br (D.C.-L.); 2 Laboratory of Microbiology, Parasitology and Hygiene, University of Antwerp, 1, 2610 Wilrijk-Antwerp, Belgium; guy.caljon@uantwerpen.be; 3 Laboratorio de Referência Nacional de Entomologia, Centro Nacional de Salud Pública, Instituto Nacional de Salud, Lima 15072, Peru; mpalominosal@gmail.com; 4 Department of Biosciences, COMSATS University, Islamabad 45550, Pakistan; rafitipu960@gmail.com; 5 Pest and Environmental Adaptation Research Group, Bio21 Institute, School of BioSciences, The University of Melbourne, 3052 Melbourne, Australia; ary@unimelb.edu.au; 6 Laboratório de Bioquímica de Insetos e Parasitos, Instituto de Biofísica Carlos Chagas Filho, Universidade Federal do Rio de Janeiro, Rio de Janeiro 21040-900, Brazil; anabahia@biof.ufrj.br; 7 Bernhard Nocht Institute for Tropical Medicine, 20359 Hamburg, Germany

**Keywords:** *Aedes aegypti*, vectorial capacity, dengue, mosquito-virus interaction, survival, fecundity, vector competence

## Abstract

**Simple Summary:**

The *Aedes aegypti* mosquito is distributed throughout several tropical countries and is considered one of the most important species vectoring arboviruses such as dengue. It has been debated whether local adaptations affect mosquito vectorial capacity. In this study, we used a network of collaborators to access *Ae. aegypti* mosquitoes from four different countries: Australia (AUS), Brazil (BRA), Pakistan (PAK), and Peru (PER). For each population, we generated a DENV-exposed group and an unexposed control and estimated survival rate and fecundity as a consequence of DENV-1 infection. Overall, DENV-1 infection reduced mosquito survival rates, but this effect was not observed in PER *Ae. aegypti* females. The number of eggs laid by *Ae. aegypti* females was similar among tested populations, with the exception of PAK in which DENV-infected mosquitoes laid fewer eggs than the uninfected control. Taken together, our results suggest geographic variation among mosquitoes that could impact dengue transmission patterns across natural settings.

**Abstract:**

The mosquito *Aedes aegypti* is distributed worldwide and is recognized as the primary vector for dengue in numerous countries. To investigate whether the fitness cost of a single DENV-1 isolate varies among populations, we selected four *Ae. aegypti* populations from distinct localities: Australia (AUS), Brazil (BRA), Pakistan (PAK), and Peru (PER). Utilizing simple methodologies, we concurrently assessed survival rates and fecundity. Overall, DENV-1 infection led to a significant decrease in mosquito survival rates, with the exception of the PER population. Furthermore, infected *Ae. aegypti* from PAK, the population with the lowest infection rate among those tested, exhibited a noteworthy reduction in egg laying. These findings collectively suggest that local mosquito-virus adaptations may influence dengue transmission in endemic settings.

## 1. Introduction

The *Aedes aegypti* mosquito is a vector of several arthropod-borne viruses (arboviruses) including dengue (DENV), chikungunya (CHIKV), Zika (ZIKV), and yellow fever (YFV) [1]. This species is originally from the African continent, but it is currently found worldwide in tropical and subtropical regions [2]. This places almost half of the global human population at risk of arbovirus transmission [3]. Mosquito-borne viruses have been on the rise globally in the past decade due to manifold reasons including favorable environmental and climatic conditions for the geographic expansion of *Ae. aegypti*, disordered urban settlements, the absence of a widely spread vaccine, and lack of timely vector control interventions [4,5,6].

Dengue transmission is influenced by the parameters governing *Ae. aegypti* vectorial capacity (VC), which represents the ability of an insect vector to transmit a given pathogen [7,8,9]. Vectorial capacity is defined by a formula describing the total number of potentially infectious bites that would eventually arise from all the mosquitoes biting a single perfectly infectious (i.e., all mosquito bites result in infection) human on a single day [10,11]. It can be expressed mathematically through vector traits directly related to transmission, such as mosquito density, susceptibility to infection (vector competence), biting rate, survival probability, and the pathogen extrinsic incubation period (EIP) [7,12]. Therefore, a detailed understanding of arbovirus epidemiology depends upon a thorough knowledge of how these life-history traits affect *Ae. aegypti* VC. Additionally, little is known about how the arbovirus infection itself influences these epidemiologically relevant parameters, with most of the data available so far focused on *Ae. aegypti* and DENV interaction [13,14].

Like other arboviruses, DENV can invade the mosquito brain and promote alterations in the mosquito’s physiology, metabolism, and behavior, thereby changing vectorial capacity and lately the pattern of disease transmission [15,16]. Therefore, estimates of traits such as mortality, longevity, and fecundity of *Ae. aegypti* need to consider their virus status. As far as we are aware, most VC estimations assume that there is little or no effect of DENV infection on these parameters [9,13]. However, some ecological aspects of the interaction between DENV and *Ae. aegypti* have been explored, pointing in general to minor to moderate fitness costs on mosquitoes due to infection [17]. For instance, Brazilian mosquitoes challenged with DENV have lower survival rates and longevity when compared to those uninfected mosquitoes [18,19].

Considering that *Ae. aegypti* has a worldwide distribution and vector competence outcomes are determined by the specific pairing of the mosquito population and the virus strain [20,21], we investigated whether the effects of DENV infection on mosquitoes can vary among populations. Here, we simultaneously investigated and compared the effects of oral DENV infection on the survival and fecundity of *Ae. aegypti* populations from four countries—Brazil, Peru, Pakistan, and Australia—which are likely to be genetically distinct [22,23]. We highlight how natural *Ae. aegypti* populations can behave differently when infected with DENV.

## 2. Materials and Methods

### 2.1. Mosquitoes

For DENV infections and fitness cost assessments, we used four *Ae. aegypti* populations from: Rio de Janeiro, Brazil (F1, BRA); Lahore, Pakistan (F9, PAK); Cairns, Australia (F3, AUS); and Campo Verde, Peru (F9, PER). *Aedes aegypti* mosquitoes were sampled using ovitraps composed of a black plastic pot with ~300 mL of piped water and either a wooden paddle (BRA, PAK, PER) or a 15-cm long strip of red felt for egg-laying (AUS) [24,25,26]. Ovitraps were installed in infested areas of the aforementioned cities in numbers to ensure the sampling represents the local genetic diversity [27]. Based on previous studies on *Ae. aegypti* population genetics across the globe using microsatellite and SNP loci data, we assumed that these mosquitoes belong to two distinct genetic clusters. The genetically distinct Pakistan and Australian mosquitoes are grouped into the Asian genetic cluster. Peruvian and Brazilian mosquitoes are grouped into the South American cluster, and although genetic information is lacking for the former population, it is highly probable they are likely distinct since all analyzed *Ae. aegypti* populations from South America (Brazil, Colombia, Venezuela, and Argentina) were clearly separated [22,28]. 

Mosquitoes from all populations were maintained in the same insectary under identical conditions. Eggs were hatched in plastic basins separated for each population containing 3 L of water and yeast extract. After this, the larvae were divided into basins containing 3 L of water, with 500 larvae per basin, and fed with TetraMin^®^ (Tetra Company, Melle, Germany). After molting into adult mosquitoes, adults were maintained in BugDorm-1 Insect Cages (30 cm × 30 cm × 30 cm) under the insectary conditions of 75±5% relative humidity and a temperature of $27\pm 2^{\;\circ }$C, in approximately 12–12 h (h) light-dark photoperiodic cycles. Mosquitoes were kept in two independent cages per population, which were later assigned to DENV-infected and -uninfected groups. Adult mosquitoes were allowed to mate and were fed *ad libitum* with a 10% sugar solution until 48 h before DENV infection.

### 2.2. Virus Strain

The infections were performed using a DENV-1 strain isolated in 2015 in Contagem, Minas Gerais State in the southeast of Brazil from a human patient (*H. sapiens*/Brasil/MV09/2015) [29]. We used C6/36 cells grown in L15 medium with 5% Fetal Bovine Serum (FBS). Infections were performed using a virus stock with a final titer of 8 × 10^6^ FFU/mL.

### 2.3. DENV-1 Challenging

About 4–5 days after emergence, adult female mosquitoes were collected from the lab colony cages and separated into small (12 cm height, 15 cm diameter) cages and deprived of food for approximately 48 h until infection. Females were orally challenged with the DENV-1 virus 6–7 days after emergence through an artificial feeding apparatus (Hemotek, Great Harwood, UK) covered with pig intestine membrane. Infective blood meal preparation was performed by adding 1 mL of virus to 1 mL of washed human erythrocytes. Preparation of the blood meal for the controls was performed by adding 1mL of culture media free of virus to 1mL of washed human erythrocytes. Mosquitoes were fed with their respective bloodmeals at a temperature of 37 °C for approximately 30 min.

### 2.4. Infection Rate Assay

Before performing the experiments investigating the effect of DENV-1 on *Ae. aegypti* survival and fecundity, a pilot assay was carried out to assess the vector competence of the four Ae. aegypti populations to this DENV-1 strain. Virus challenging was performed as described above and 40 fully engorged females per population were separated into small cages. At 14 days post-infection (dpi), mosquitos were frozen and stored at −80 °C. Dengue virus infection was checked by extracting RNA from whole mosquitoes using the QlAamp Viral RNA Mini kit (Qiagen, Hilden, Germany) following the manufacturer’s instructions. Detection and quantification of viral RNA was performed using qRT-PCR with SuperScript^TM^ III Platinum^TM^ One-Step qRT-PCR Kit (Thermo Fisher Scientific, Invitrogen, Waltham, MA, USA) in QuantStudio 6 Flex Real-Time PCR System (Applied Biosystems, Waltham, MA, USA), as previously described [30,31]. Viral copy numbers were calculated by interpolation onto an internal standard curve made up of a six-point dilution series (10^1^–10^6^ FFU/mL) of DENV-1 [29].

### 2.5. Survival and Fecundity Assessments and Wing Length

Fully engorged females fed on infectious or control blood were individualized into labeled cylindrical plastic vials (6.5 cm height and 2.5 cm diameter), which contained moistened cotton with filter paper on it as an oviposition substrate and closed with mosquito netting. Sugar feeding was performed by daily placing small balls of cotton containing a 10% sucrose solution on the top of the vials. Survival was monitored daily until all the mosquitoes died. Dead mosquitos were removed from the plastic vials and wing length was measured as the distance from the axillary incision to the apical margin, excluding the fringe [32]. This measurement was performed as a measure of mosquito body size as we are aware that it might influence insect survival and fecundity [14,18,19,33,34,35].

Fecundity consisted of the total number of eggs laid by a female mosquito during its lifetime [19]. DENV-uninfected bloodmeals were offered weekly using the feeding apparatus described above. Around 3–4 days later the filter paper was removed from the vial and the number of eggs was counted and recorded.

### 2.6. Statistical Analysis

In the pilot vector competence assay, the DENV load was compared among *Ae. aegypti* populations using the Kruskal–Wallis rank sum test. A Kaplan–Meier (KM) survival curve was made for all populations together in order to examine the general effect of DENV exposure on mosquito survival. Then, KM curves were made for each mosquito population (AUS, BRA, PAK, PER), according to treatments (DENV-exposed or uninfected), and a log-rank test was performed to examine virus effects on mosquito survival. The significance level was corrected for multiple comparisons using the Bonferroni criteria. Moreover, survival and DENV infection associations were also analyzed using a Cox proportional hazard regression analysis, in which treatment and wing length were included as covariates. The Mann–Whitney–Wilcoxon test for non-normal distribution and continuity corrections was performed. All data analysis was performed in the R environment [36].

## 3. Results

### 3.1. Infection Rate Assay

Infection rates were calculated at 14 dpi for each population using 40 mosquitoes. The higher DENV infection rate was observed for PER (95%) and the lowest one for PAK (47.5%). AUS and BRA had intermediate infection rates: 82.5 and 77.5%, respectively. 

**Figure 1:**
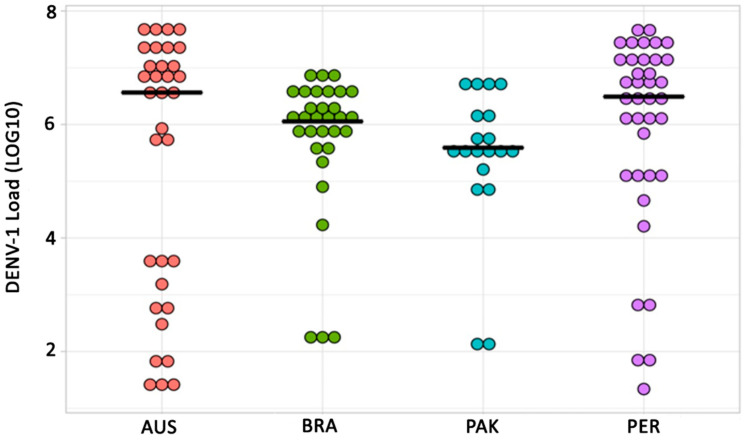
DENV-1 copies per population. Each dot represents a DENV-1 infected mosquito. No statistical difference in DENV-1 load was found among the populations (H = 6.1, df = 3, *p*-value = 0.107). The dark line represents the median DENV-1 load per population.

However, no statistically significant differences in DENV-1 loads were observed across the infected *Ae. aegypti* mosquito populations (H = 6.1, df = 3, *p*-value = 0.107) (Figure 1).

### 3.2. Survival

**Figure 2:**
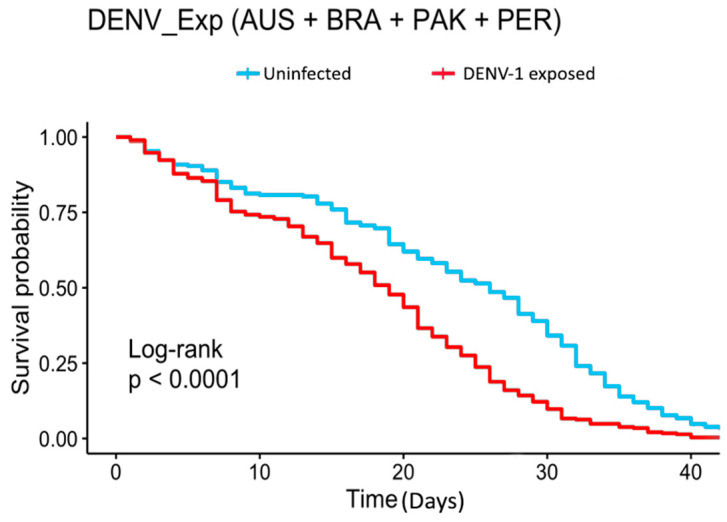
Kaplan–Meier survival curves depicting the survival probability of control (blue) and DENV-exposed mosquitoes (red).

**Figure 3:**
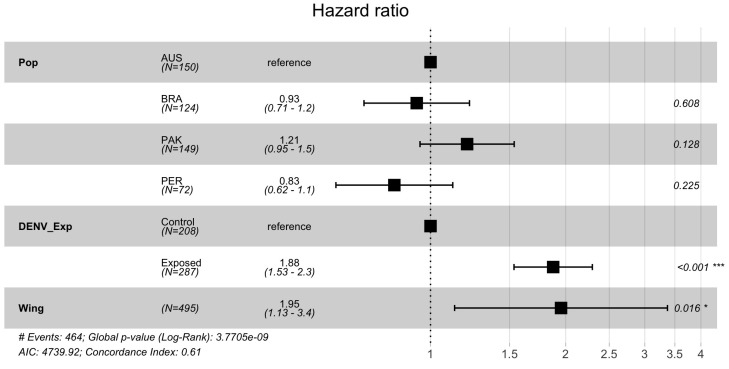
Cox proportional hazard ratios. Pop: comparing mortality among populations with AUS as reference; DENV_Exp: showing a significant cumulative risk increase for the event of death to occur in individuals exposed to DENV-1 as opposed to the control populations. Wing: demonstrating a significant increase in the event of death in larger mosquito individuals. Error bars represent a 95% confidence interval. N = Number of mosquitos used, AUS = Australia; BRA = Brazil; PAK = Pakistan; PER= Peru. *p < 0.05; *** *p* < 0.001.

**Figure 4:**
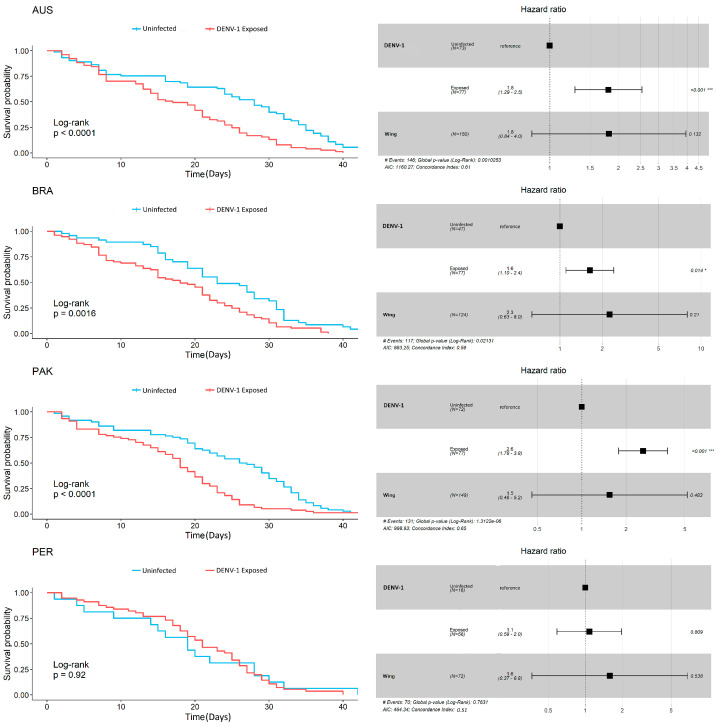
Survival analysis and Cox regressions by population. Left figures indicate survival probability over time (days) with log-rank *p*-values, right figures show the corresponding Cox models with the respective hazard ratio for treatments and wing length. In the infection status, the control group was used as a reference. Wing length was used as a covariate but showed no influence on mosquito mortality for the populations. Note that the cumulative hazard for the event of death to occur did not rise significantly for PER mosquitoes, differently from the other populations tested. Error bars represent a 95% confidence interval. N = Number of mosquitos used. AUS = Australia; BRA = Brazil; PAK = Pakistan; PER = Peru. * *p* < 0.05; *** *p* < 0.001.

**Figure 5:**
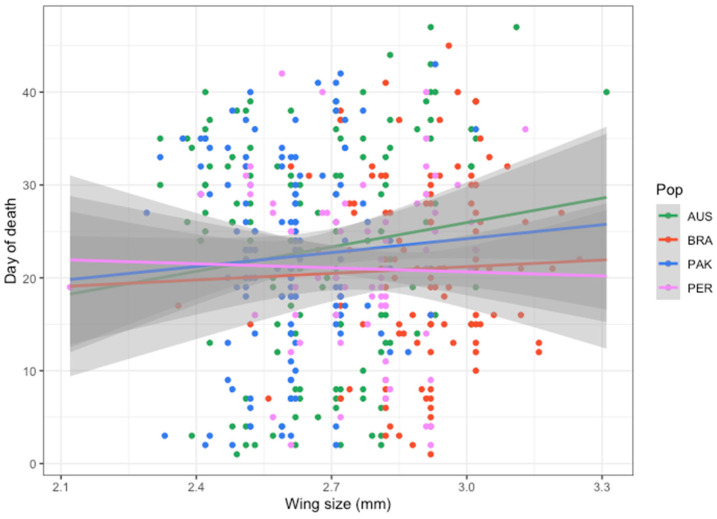
Linear regression showing the effect of wing size on *Ae. aegypti* mortality from the four tested populations. The shaded area represents the 95% confidence level interval for predictions from a linear model. AUS = Australia; BRA = Brazil; PAK = Pakistan; PER = Peru; Pop = Population.

We monitored the survival rate of 495 *Ae. aegypti* mosquitoes, 287 dengue-exposed and 208 from the negative control. Overall, DENV-1 infection negatively affected *Ae. aegypti* survival rates since uninfected insects had lower mortality over time than their exposed counterparts. The divergence between survival curves of exposed and uninfected mosquitoes started around one week after the infective/control blood meal (Figure 2, Log-rank: *p* < 0.0001). The multivariate Cox model in Figure 3 confirmed this result, showing a 1.88 times increase in the chance of the event of death for DENV-1 exposed mosquitoes, disregarding insect population (*p* < 0.001). When comparing the treatments (uninfected or exposed) within populations, the KM survival curves demonstrated significant differences between exposed and control mosquitoes from AUS (log-rank: *p* < 0.0001), BRA (log-rank: *p* = 0.0016) and PAK (log-rank: *p* < 0.0001), which were reinforced by the Cox models (Australia HR = 1.8, Brazil HR = 1.6, Pakistan HR = 2.6, all showing significant *p* values (Figure 4). For Peru, no significant difference was seen between the treatments for the Kaplan-Meier curves, nor for the Cox model. Wing length had a controversial influence on survival. If data from all mosquito populations are analyzed together, we see an effect of size on survival (Figure 3). However, if we break down the analysis considering the mosquito population, we observe no detectable effect of wing length for the populations (Figure 4). We observed a tendency for larger mosquitoes to survive longer than smaller insects in all populations, but the effects of wing size on mortality were not statistically significant (Figure 5).

### 3.3. Fecundity

**Figure 6:**
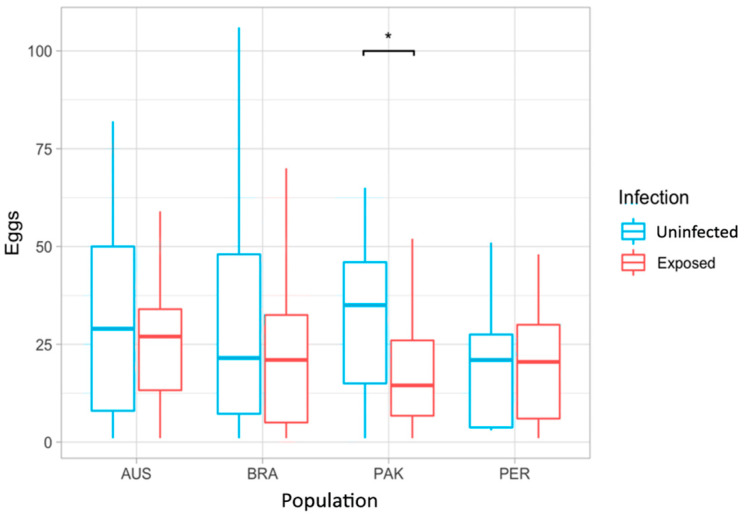
Box plot of fecundity comparing all females in a population with each other and infection status as well as with females in other populations. The only population where we saw a significant difference between status was the Pakistan population (W = 468, *p*-value = 0.0167). AUS = Australia; BRA = Brazil; PAK = Pakistan; PER = Peru; Pop= Population. The asterisk represents a *p* < 0.05.

Overall, 153 *Ae. aegypti* females laid at least one egg during their lifetime, representing 31.03% of the 493 female mosquitoes involved in the study. Considering only those that laid eggs, an average of 34.88 eggs per female was recorded. Of the 153 that laid eggs, 79 (51.6%) were from the control group. However, an uneven distribution of egg-laying females was observed among populations: 52 from AUS (35.1%), 34 from BRA (27.6%), 45 from PAK (30.2%), and 22 from PER (30.1%). Box plot and Mann–Whitney–Wilcoxon tests showed a significant difference among treatments only for the PAK population, in which case DENV-infected mosquitoes laid significantly fewer eggs than DENV-uninfected (Figure 6, W = 468, *p*-value = 0.0167).

## 4. Discussion

In most dengue-endemic countries, *Ae. aegypti* is classified as the dengue primary vector, highlighting the high propensity of this mosquito species to vector arboviruses to humans on a myriad of local specific conditions in regards to seasonal temperature, human dwelling’s structure, breeding site types and availability, rainfall regime, and local mosquito-virus interactions. Herein, we report that mosquitoes from different countries might vary when infected with a single DENV isolate. Using *Ae. aegypti* mosquitoes originating from four different countries, Australia (AUS), Brazil (BRA), Pakistan (PAK), and Peru (PER) we found that PAK had a lower infection rate for the DENV-1 isolate used, but the viral copies did not differ among populations. The infected mosquitoes from AUS, BRA, and PAK showed a reduction in their survival compared to their uninfected counterparts, whereas the survival rate of PER *Ae. aegypti* females was not affected by DENV-1 infection.

The DENV isolate we used in the assays was isolated in 2015 in the city of Contagem, in the metropolitan region of Belo Horizonte, southeast Brazil. Most likely, this DENV-1 isolate belongs to genotype V, clade I [37]. This DENV-1 strain is spread throughout South America, having been detected in the last decades in the Northern and Southern regions of Brazil, Paraguay, Colombia, and Venezuela [37,38,39,40,41]. Therefore, mosquito populations from South America have probably experienced a closer evolutionary history with this virus than more distant *Ae. aegypti* populations. We therefore hypothesized that BRA and PER populations might be less affected by infection with this DENV-1 genotype, whereas AUS and PAK would be most affected. Our results are only in part agreement with this hypothesis. Exposed mosquitoes from the PER population were the only ones not negatively affected by this virus, while PAK DENV-exposed females were the only ones to show a reduction in egg laying after blood meal.

Geographic variation of mosquito traits has previously been explored in vector competence studies by collecting them and challenging them with different microorganisms in laboratory-controlled assays [42,43,44,45,46]. Public health authorities can then estimate the impact of the target microorganism across the country. Variation in arboviruses vector competence is not fully understood but has been mapped to quantitative trait loci (QTL) [21,47]. Additionally, variation in *Ae. aegypti* susceptibility can be affected by specificities of the mosquito-virus strain combination tested, which is known as genotype-by-genotype (G × G) interactions [20,21]. Several papers show the existence of G × G interactions regulating DENV susceptibility in *Ae. aegypti* [20,48,49,50,51], as well as hypothesizing its role in explaining infection phenotypes with other arboviruses [14,45,52]. We performed a pilot study with our four mosquito populations aiming to confirm their infection when challenged with the same DENV-1 isolate. Although it might be appealing to infer G × G interactions to explain the reason why PAK mosquitoes had a lower infection rate, we recognize our assay was not designed to specifically investigate G × G interactions, which would require more viral strains. 

One important limitation of our study, especially when testing vector competence, was the absence of screening DENV-1 load in saliva. Vector competence is defined as the physiological ability of a mosquito to become infected with and transmit arboviruses [53]. The most appropriate method to assess the vector competence of a mosquito species is to evaluate the presence of arbovirus in saliva because it means the virus is able to pass through the midgut infection barrier and the salivary gland infection barrier, two common bottlenecks of viral copies and diversity [54,55]. The midgut infection barrier results from either the inability of the virus to enter the midgut cells due to the absence of suitable receptors, and/or the inability of the virus to replicate within the midgut cells [56]. In parallel, the salivary gland infection barrier occurs when the virus fails to infect the cells of the salivary gland. As an example of this type of work, five *Ae. albopictus* populations from Spain have similar infection rates, when Zika virus (ZIKV) is assessed over whole mosquito bodies, suggesting that the virus has overcome the midgut infection barrier; however, saliva data showed only a few specimens had ZIKV in their saliva, highlighting the effectiveness of the salivary infection barrier in these populations [57].

Mosquito survival is among the most important traits usually measured to determine the vectorial capacity of a population [58]. In theory, the longer a mosquito lives, the greater the number of opportunities a host-seeking female has to find a host to blood feed. The *Ae. aegypti* survival rate is among the most affected traits after arbovirus infection [13,18,19]. Previous papers have speculated that the high mortality of the infected group was due to an intense immune activation due to infection. When eliminating a viral infection, an immune response may create a biological burden that consequently increases mortality, as has been described for other insect–pathogen systems [19,59,60]. The cellular and humoral immunity responses of *Ae. aegypti* mosquitoes to arboviruses such as DENV may use RNA interference, a major component of the mosquito’s innate immune response, to modulate infection by producing molecules that inhibit virus replication [61,62,63]. Taking all mosquitoes together, DENV-1 presented a negative effect on *Ae. aegypti* survival rates, reducing mosquito lifespan, with the exception of PER. We note that the sample size of uninfected PER controls was low though survival curves of DENV-infected and uninfected mosquitoes from PER support similar patterns of infected and control individuals. Regarding the wing length, we observed that the Cox proportional hazard rate showed an increase in mosquito mortality associated with mosquito size (Figure 3), a trend also observed when data were classified according to mosquito population (Figure 5) although not statistically significant. The increasing mortality with mosquito size is surprising since previous data from laboratory-controlled experiments or mark-release-recapture revealed smaller mosquitoes tend to have shorter lifespans than larger individuals [64,65,66,67,68]. It is assumed that larger mosquitoes emerge from the breeding sites with more energy reserves than smaller individuals, which decreases the need for continuously seeking blood feed or sugar-based feeding for metabolism [64,65,69,70]. Although consistent among all populations, we speculate that this unexpected observation is most likely due to rearing conditions rather than biological reasons.

Another trait that is often evaluated in infected mosquitoes is female fecundity, i.e., the number of eggs laid. Dengue virus had an age-dependent effect on mosquito fecundity since infected females laid fewer eggs per clutch than uninfected controls in the third and subsequent oviposition cycles [18,19,71]. *Aedes aegypti* females infected intra-thoracically with DENV-2 also had an increase of up to 50% in their locomotor activity when compared to uninfected control mosquitoes [72]. Ideally, fecundity data is reported by assessing egg laying per gonotrophic cycle [18,19,73,74]. However, considering only 31.03% of the *Ae. aegypti* females involved in the study laid eggs at least once, we summed numbers across oviposition cycles. The PAK population exhibited a prominent effect of DENV infection on fecundity, with exposed individuals having lower egg laying than uninfected insects. We note that the PAK population had the lowest infection rate among the tested populations, and perhaps the fecundity costs due to infection reflect exposed mosquitoes that screened as negative, with fitness effects reflecting the activation of an immune response pathway caused by the arbovirus infection [18,19,60]. One limitation of our summation approach across different oviposition cycles is that we are not able to determine age-dependent effects caused by DENV infection. The effects of DENV in *Ae. aegypti* fecundity tend to be more intense over time, especially around 14 days post-infection, i.e., at the second oviposition cycle [71]. Finally, an additional limitation is that we have not evaluated egg fertility, an important parameter for *Ae. aegypti* biology that could provide additional insights across populations. 

## 5. Conclusions

This manuscript provides information on the fitness costs in terms of survival rate and fecundity of four different *Ae. aegypti* populations when challenged with a DENV-1 isolate. Mosquito populations behaved differently when infected with a DENV-1 isolate, suggesting local mosquito-virus interactions could play a role in dengue transmission in different geographic settings.

## Data Availability

The raw data supporting the conclusions of this article will be made available by the authors upon request.
